# Sex- and age-specific association between outdoor light at night and obesity in Chinese adults: A national cross-sectional study of 98,658 participants from 162 study sites

**DOI:** 10.3389/fendo.2023.1119658

**Published:** 2023-02-20

**Authors:** Xiaoyun Zhang, Ruizhi Zheng, Zhuojun Xin, Zhiyun Zhao, Mian Li, Tiange Wang, Min Xu, Jieli Lu, Shuangyuan Wang, Hong Lin, Weiqing Wang, Guang Ning, Yufang Bi, Yuhong Chen, Yu Xu

**Affiliations:** ^1^Department of Endocrine and Metabolic Diseases, Shanghai Institute of Endocrine and Metabolic Diseases, Ruijin Hospital, Shanghai Jiaotong University School of Medicine, Shanghai, China; ^2^Shanghai National Clinical Research Center for Metabolic Diseases, Key Laboratory for Endocrine and Metabolic Diseases of the National Health Commission of the PR China, Shanghai Key Laboratory for Endocrine Tumor, State Key Laboratory of Medical Genomics, Ruijin Hospital, Shanghai Jiaotong University School of Medicine, Shanghai, China

**Keywords:** outdoor light at night, circadian system, obesity, sex difference, age

## Abstract

**Background:**

Exposure to light at night (LAN) is a potent disruptor of the circadian system. Whether LAN exposure exerts a sex- or age-specific influence on obesity needs investigation.

**Objectives:**

To estimate the sex- and age-specific associations of exposure to outdoor LAN and obesity based on a national and cross-sectional survey.

**Methods:**

The study included a nationally representative sample of 98,658 adults aged ≥ 18 years who had lived in their current residence for ≥ 6 months from 162 study sites across mainland China in 2010. Outdoor LAN exposure was estimated from satellite imaging data. General obesity was defined as body-mass index (BMI) ≥ 28 kg/m^2^ and central obesity was defined as waist circumference ≥ 90 cm in men and ≥ 85 cm in women. Linear and logistic regression models were used to examine the associations between LAN exposure and prevalent obesity in sex and age categories.

**Results:**

A monotonically increasing association of outdoor LAN with BMI, waist circumference was observed in all sex and age categories, except for adults aged 18-39 years. Significant associations of LAN exposure with prevalent obesity were observed in each sex and age category, especially in men and older people. Per 1-quintile increase in LAN was associated with 14% increased odds of general obesity in men (odds ratio, OR=1.14, 95% confidence interval, CI=1.07-1.23) and 24% in adults aged ≥ 60 years (OR=1.24, 95% CI=1.14-1.35). Per 1-quintile increase in LAN was associated with 19% increased odds of central obesity in men (OR=1.19, 95% CI=1.11-1.26) and 26% in adults aged ≥ 60 years (OR=1.26, 95% CI=1.17-1.35).

**Conclusions:**

Increased chronic outdoor LAN exposure was associated with increased prevalence of obesity in sex- and age- specific Chinese populations. Public health policies on reducing light pollution at night might be considered in obesity prevention.

## 1 Introduction

The endogenous circadian timing system and physiological functions have developed under a periodicity of approximately 24-hour rhythm adapted to the external light-dark cycle through the evolution process in humans. Over the past centuries, the increasing level of urbanization in modern cities has led to the continuously expanding use of light at night (LAN), especially in the urban area, which is also recognized as light pollution that poses a threat to human health and the natural environment (1–4). Growing evidence has demonstrated the deleterious effects of LAN exposure on multiple adverse outcomes including obesity, metabolic disorders, mental disorders, cancer, and cardiovascular disease (5–8). The potential pathological mechanisms might include circadian disruption, sleep deprivation, and suppression of melatonin secretion (9).

Animal studies have revealed that even dim light at night (as little as 5 lx) was associated with altered feeding behaviors, metabolic dysfunction, and increased weight gain (10, 11). In humans, epidemiologic studies have shown that exposure to LAN contributed to obesity independent of other confounders, although mainly in cross-sectional designed studies or in a single middle-aged or elderly population (5).

During the past decades, obesity has increasingly become a major health issue in China, posing a substantial burden on societal health (12). Our previous study showed that compared with people of other ethnicities, Chinese adults were more likely affected by the detrimental effects of obesity, and sex difference also played a part (13). In Asian populations, women are more likely to accumulate percutaneous adipose tissue while men accumulate visceral adipose tissue (14). In the Chinese population, sex difference in body composition such as lean mass and fat mass existed throughout the lifespan, and age-related changes in body composition presented differently in men and women (15, 16). Moreover, melatonin, the critical hormone controlled by the master circadian pacemaker located in the hypothalamic supra-chiasmatic nucleus, was thought to decrease with age (17). Therefore, whether LAN exposure exerts a sex- or age-specific influence on obesity prevalence in Chinese adults needs investigation. Age groups were categorized into young or early adulthood (18-39 years), middle adulthood (40-59 years), and older adulthood (60+ years) (18).

## 2 Materials and methods

### 2.1 Study design and participants

The China Noncommunicable Disease Surveillance 2010 was a cross-sectional study and conducted based on the National Disease Surveillance Point System of the Chinese Center for Disease Control and Prevention (CDC). A nationally representative sample of the general population aged ≥ 18 years was selected from 162 study sites covering the major geographic areas of all 31 provinces, autonomous regions, and municipalities in mainland China. Details of information on the study population, design, and protocol have been described previously (19–22). Briefly, a complex multistage probability sampling design was implemented at each study site to enroll adults who had lived in their current residence for at least 6 months. At the first step of sampling, 4 subdistricts at each site were selected with probability proportional to size (PPS). At the second step, 3 neighborhood communities or administrative villages were selected with PPS. At the third step, 50 households were randomly selected from the listed households within each aforementioned neighborhood community or administrative village. At the final step, one person aged 18 years or older was selected using a Kish selection table from each household. A total of 109,023 residents were selected, and 98,658 participated in the investigation. The Ethical Review Committee of China CDC and other participating institutes approved the study protocol. All participants provided written informed consent.

### 2.2 Outdoor LAN exposure

Outdoor light at night is defined as excessive, inappropriate utilization of artificial outdoor lighting emitted by various sources, including street lighting, digital screens, residential and commercial buildings, et al. (4). The light imaging data were captured by sensors of satellites and further assessed by the US Defense Meteorological Satellite Program (DMSP) through the Operational Line-scan System (OLS). The processed DMSP-OLS imaging data were then transferred into the US National Oceanic and Atmospheric Administration (NOAA) National Geophysical Data Center (NGDC) database. Due to the high gain settings of sensors, in the original DMSP-OLS nighttime light product, bright sources can’t be accommodated leading to the over-saturation of recorded light imaging in urban centers within a limited dynamic range. Therefore, the most recent Global Radiance Calibrated Nighttime Lights combined the sparse data acquired at low and high gain settings to solve the sensor “saturation” problem (23). After excluding the other environmental light disturbers including sun and moon luminance, the light imaging data theoretically indicate the relative intensity of LAN at ground level. The recent high dynamic range data recorded from January 11, 2010 to December 9, 2010 (data version: ‘F16_20100111-20101209_rad_v4’) was downloaded from the NOAA website (https://ngdc.noaa.gov/eog/dmsp/download_radcal.html) for current analysis. LAN exposure of participants at each study site was assigned the mean nighttime radiance of the outdoor LAN of the study site, which was either a district in cities or a county in rural areas. Outdoor LAN data from satellite images of the NOAA were commonly used in literature as a proxy measure of the actual LAN exposure (7, 8, 24, 25).

### 2.3 Data collection

Based on a standard protocol, data collection was conducted by trained staff at examination centers of local health stations or community clinics in the participants’ residential areas at each study site. Information on the demographic characteristics, socioeconomic status, lifestyle factors, medication history, and history of chronic diseases was recorded by administering a comprehensive questionnaire to participants face to face by trained staff. Current smoking was defined as having smoked 100 cigarettes in one’s lifetime and currently smoking cigarettes. Current drinking was defined as alcohol intake more than once per month during the past 12 months. The Global Physical Activity Questionnaire, which contains questions about the frequency of moderate and vigorous activities and walking per week, was used to assess the level of physical activity. At least 150 min/week moderate-intensity or at least 75 min/week vigorous intensity or at least 150 min/week moderate and vigorous physical activity were defined as an active level of physical activity. The dietary habits were recorded using a food frequency questionnaire on the consumption of typical food items during the previous 12 months. A healthy diet score, recommended by the American Heart Association (26) and modified using soy protein to replace fiber intake (22), was used for analysis in the current study. Blood samples were obtained after a ≥ 10 h overnight fast, the details of which were previously described (20). The homeostasis model assessment of insulin resistance (HOMA-IR) was calculated as levels of fasting serum insulin (μU/mL) multiplying levels of fasting plasma glucose (mmol/L) divided by 22.5 (27). Comprehensive quality control and assurance measures were implemented to ensure data validity and reliability.

### 2.4 Outcome measures

Anthropometric measurements including weight and height were conducted adhering to a standard protocol. BMI was calculated as body weight in kilograms divided by body height in meters squared (kg/m^2^). Waist circumference was measured as the length of the midway between the lower edge of the costal arch and the upper edge of the iliac crest while participants were asked to take a standing position. General obesity was defined as BMI ≥ 28 kg/m^2^ and central obesity was defined as waist circumference ≥ 90 cm in men and ≥ 85 cm in women based on the criteria recommended by the National Health Commission of the People’s Republic of China (28).

### 2.5 Statistical analysis

To account for the multistage probability sampling design of the survey, the appropriate weights and design factors were considered to represent the overall Chinese adult population aged ≥ 18 years in all the analyses. Weight coefficients were derived from the 2010 China population census data and the sampling scheme of the current survey to obtain national estimates. Sex and age were key stratification factors (in addition to urban/rural areas and geographical regions) used to calculate weight coefficients. Details of weighting methods are provided in the **Supplementary Appendix**. Data were described as means (95% confidence interval, CI) for continuous variables and percentages (95% CI) for categorical variables. The ANOVA statistical method was used for comparing continuous variables and the chi-square tests for comparing categorical variables. Splines were well-fitted to capture the linear or non-linear relationships between exposure and health outcomes in the epidemiological study. We transformed the linear variable of outdoor LAN incorporating a restricted cubic spline (RCS) function of LAN with the reference value of 0.1 nW/cm^2^/sr. Three knots 5th, 50th, and 95th percentiles were automatically set for the splines, as commonly used with RCS functions (29). The estimated exposure-response curve was used to evaluate the effect of outdoor LAN exposure as a continuous variable on obesity-related parameters including BMI and waist circumference stratified by sex (men and women) and age groups (18-39 years, 40-59 years, 60+ years). In addition, the level of outdoor LAN exposure was categorized into quintiles and logistic regression analysis was conducted to assess the association between outdoor LAN exposure in quintiles and prevalence of general obesity or central obesity. The odds ratios (ORs) were calculated for quintiles 2-5 *vs.* the lowest quintile of LAN exposure or per 1-quintile increase in LAN exposure. Assumption tests of regression models were conducted and assumptions were met (**Tables S1–3**). We selected *a priori* potential confounders for adjustment in multivariable models based on knowledge of their associations with outdoor LAN exposure and obesity. They included age (in sex strata) or sex (in age strata), education, smoking status, drinking status, physical activity, healthy diet score, urban or rural areas, household income, and HOMA-IR. To test for interaction by sex or age on the association of outdoor LAN exposure with general obesity and central obesity, interaction terms were added to the unadjusted model and interactions were assessed with a likelihood-ratio test. If an interaction existed for sex or age, analyses were to be conducted, and results were to be presented separately in sex and age subgroups. Because missing values of healthy diet score (n=6128) exceeded 5%, they were imputed using fully conditional specification method with the mice package (version 3.13.0) for R (version 4.1.2), assuming missing at random. We used linear regression for the imputation and age, sex, education level, physical activity, urban or rural areas, household income and light at night were auxiliary variables without missing data. Distributions of imputed and observed values were compared, and no significant difference was observed. Missing values for other variables were not imputed because missingness was 1.4% for HOMA-IR and < 0.1% for other variables.

R version 4.1.2 (R Foundation for Statistical Computing, Vienna, Austria) was used for data analyses. A two-sided *p* value less than 0.05 was considered as statistical significance.

## 3 Results

**Figure 1:**
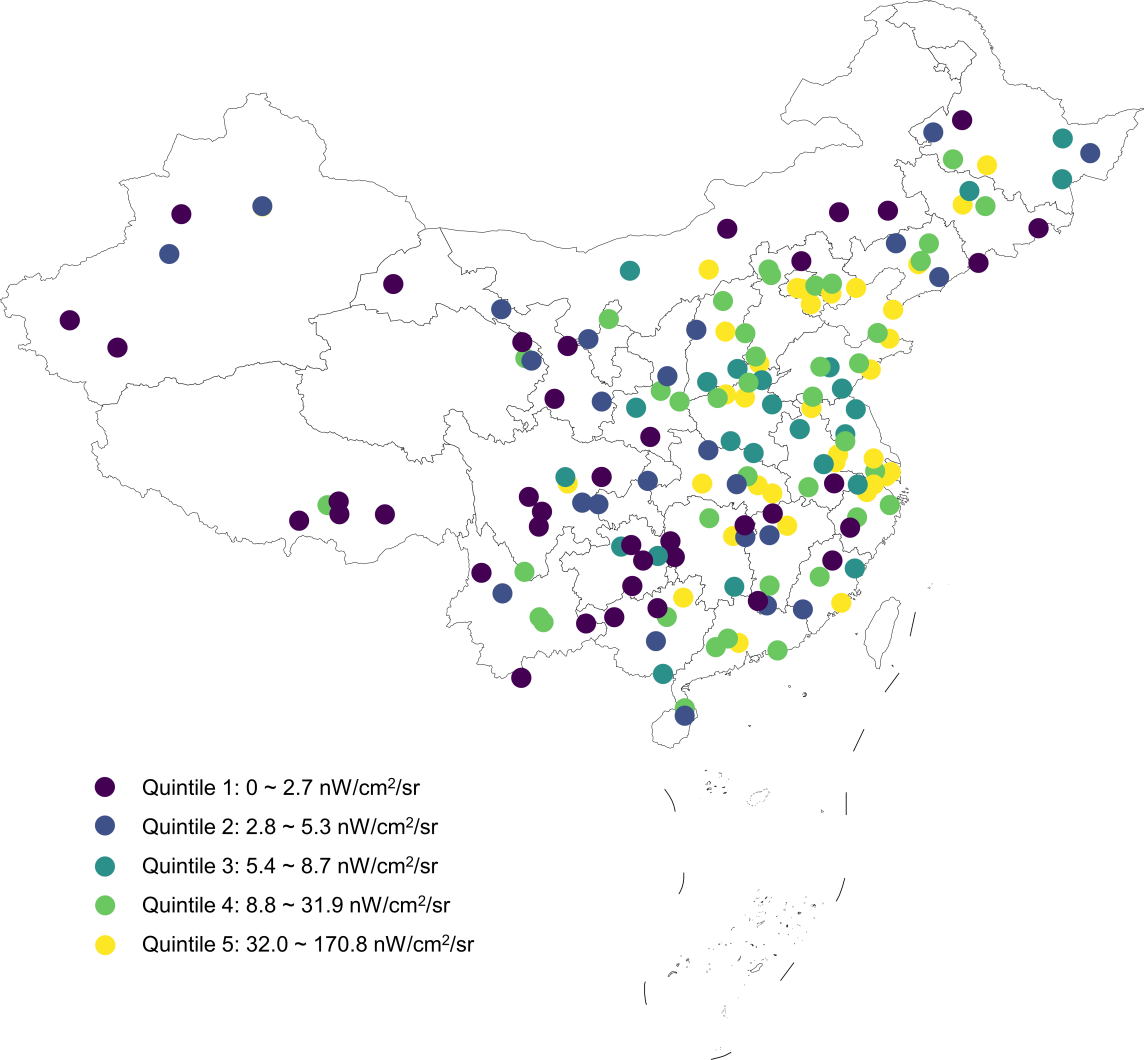
Geographical distribution of the 162 study sites in the current study with colors indicating quintiles of outdoor LAN exposure.

As shown in **Figure 1**, there were substantial differences in the outdoor LAN exposure levels among the 162 study sites across China. The study sites in the eastern region had higher levels of outdoor LAN, in contrast to those in the western region with lower levels of outdoor LAN. The frequency distribution of participants exposed to outdoor LAN is skewed to the left, as well as for those by sex and age groups (**Figure S1**). The means and 95% CIs of outdoor LAN in quintile 1 to quintile 5 were 1.4 (1.1-1.6), 4.0 (3.6-4.4), 7.1 (6.6-7.7), 17.0 (14.7-19.3), 85.0 (66.1-104.0) nW/cm^2^/sr, respectively. Participants exposed to a higher level of outdoor LAN were more likely to be older, more educated, less physically active, living in the urban area, having a higher household income and higher levels of HOMA-IR (**Table 1**).

**Table 1:** Baseline characteristics of the participants.

Characteristics	Outdoor light at night exposure (nW/cm^2^/sr)				
	Quintile 1 (0 ~ 2.7)	Quintile 2 (2.8 ~ 5.3)	Quintile 3 (5.4 ~ 8.7)	Quintile 4 (8.8 ~ 31.9)	Quintile 5 (32.0 ~ 170.8)
Number of participants	24151	14392	15028	23773	21314
Women (%)	48.5 (47.3 - 49.7)	49.1 (47.7 - 50.6)	49.0 (47.3 - 50.7)	47.9 (46.5 - 49.2)	51.3 (49.2 - 53.4)
Age (year)	39.9 (38.6 - 41.2)	43.5 (41.6 - 45.5)	43.6 (42.1 - 45.1)	42.2 (41.0 - 43.4)	44.0 (42.4 - 45.6)
Age category (%)					
18-39 years	53.6 (49.7 - 57.5)	42.8 (37.3 - 48.4)	44.3 (40.4 - 48.2)	45.9 (42.4 - 49.5)	43.1 (37.9 - 48.3)
40-59 years	33.9 (30.9 - 36.9)	41.1 (37.4 - 44.8)	36.4 (34.2 - 38.5)	39.7 (37.1 - 42.2)	39.0 (35.3 - 42.7)
≥ 60 years	12.5 (11.2 - 13.9)	16.1 (12.9 - 19.2)	19.3 (16.5 - 22.1)	14.4 (12.6 - 16.2)	17.9 (15.4 - 20.3)
Senior middle school or higher (%)	16.1 (12.0 - 20.2)	20.4 (17.2 - 23.6)	18.2 (14.1 - 22.3)	27.5 (21.5 - 33.5)	48.5 (41.7 - 55.2)
Higher level of physical activity (%)	87.8 (84.4 - 91.3)	83.3 (80.4 - 86.3)	82.3 (78.2 - 86.4)	75.4 (70.5 - 80.4)	81.5 (78.4 - 84.6)
Healthy diet score ≥ 3 (%)	8.6 (5.8 - 11.3)	17.2 (12.6 - 21.8)	14.9 (10.0 - 19.8)	17.4 (13.1 - 21.6)	31.8 (25.9 - 37.6)
Current smoking (%) ^†^	30.2 (26.9 - 33.5)	28.4 (25.9 - 30.9)	27.9 (25.5 - 30.3)	28.6 (26.6 - 30.5)	26.4 (24.0 - 28.8)
Current drinking (%) ^†^	29.6 (24.7 - 34.5)	29.7 (26.0 - 33.3)	29.0 (25.5 - 32.5)	29.0 (26.2 - 31.8)	30.2 (27.8 - 32.7)
Household income (10000 Chinese Yuan)	1.9 (1.7 - 2.2)	2.2 (1.7 - 2.6)	2.3 (1.8 - 2.9)	2.8 (2.5 - 3.0)	4.1 (3.8 - 4.4)
Living in urban area (%)	20.3 (5.5 - 35.0)	29.6 (7.8 - 51.4)	31.9 (11.2 - 52.5)	66.5 (48.3 - 84.7)	88.6 (72.6 - 100.0)
HOMA-IR ^†^	1.7 (1.5 - 1.8)	1.6 (1.5 - 1.7)	1.6 (1.5 - 1.7)	1.8 (1.7 - 1.9)	2.0 (1.9 - 2.1)
BMI (kg·m^-2^) ^†^	23.1 (22.8 - 23.4)	23.3 (23.0 - 23.6)	23.7 (23.3 - 24.0)	24.1 (23.8 - 24.5)	24.2 (23.8 - 24.6)
Waist circumference (cm) ^†^	78.1 (76.8 - 79.3)	78.9 (77.9 - 80.0)	80.1 (78.9 - 81.3)	81.9 (80.9 - 82.8)	82.1 (80.9 - 83.2)
WHtR ^†^	0.49 (0.48 - 0.50)	0.49 (0.49 - 0.50)	0.50 (0.49 - 0.50)	0.50 (0.50 - 0.51)	0.50 (0.50 - 0.51)
General obesity (%) ^‡^	9.2 (7.6 - 10.8)	9.5 (7.8 - 11.2)	11.7 (9.9 - 13.6)	14.2 (12.1 - 16.3)	15.0 (12.6 - 17.5)
Central obesity (%) ^‡^	18.0 (14.8 - 21.3)	19.7 (16.5 - 22.8)	24.0 (20.1 - 27.9)	28.7 (25.5 - 31.9)	30.8 (27.1 - 34.5)

Data are shown as means or percentages with 95% confidence intervals, unless indicated otherwise.

^†^ There were 4 missing values for current smoking, 9 for current drinking, 1381 for HOMA-IR, 85 for BMI, 75 for waist circumference and 85 for WHtR.

^‡^ General obesity was defined as BMI ≥ 28 kg/m^2^. Central obesity was defined as waist circumference ≥ 90 cm in men and waist circumference ≥ 85 cm in women.

HOMA-IR, homeostasis model of insulin resistance; BMI, body-mass index; WHtR, waist-to-height ratio.

Generally, the estimated prevalence of general obesity and central obesity increased across quintiles of outdoor LAN exposure in the overall study population and in sex and age groups (**Table S4**). Because significant interactions of sex and age for the association between outdoor LAN exposure and obesity were observed (all *P*_interaction_ < 0.01), subsequent results were presented in sex and age groups separately.

**Figure 2:**
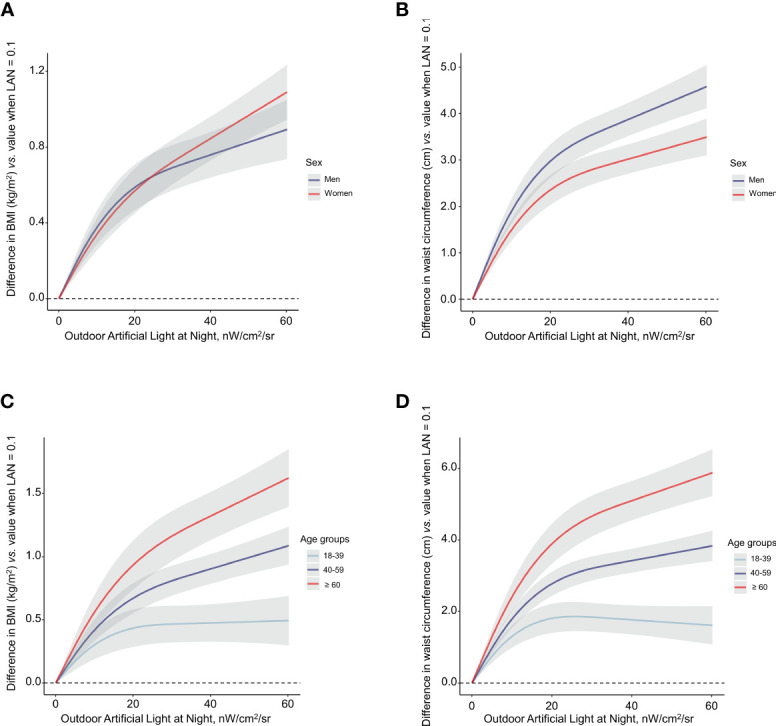
Associations of outdoor LAN exposure with BMI **(A, C)** and waist circumference **(B, D)** categorized by sex and age. Light at night was fitted as a smooth term using a restricted cubic spline with 3 knots. The reference outdoor LAN exposure was 0.1 nW/cm^2^/sr. Shading indicates 95% confidence interval. Participants within the top right 10% of outdoor LAN exposure were trimmed for the spline model. The model was adjusted for age (for sex strata), sex (for age strata), education, smoking status, drinking status, physical activity, healthy diet score, urban or rural areas, household income, and HOMA-IR. BMI, body-mass index; HOMA-IR, homeostasis model of insulin resistance.

**Figure 2** showed the exposure-response curve for outdoor LAN exposure levels with BMI and waist circumference by sex and age groups, respectively. A monotonically increasing dose-dependent relationship was consistently found between outdoor LAN exposure with BMI and waist circumference in both men and women after adjustment (**Figures 2A, B**). This was also found in participants aged 40-59 years or ≥ 60 years. However, in participants aged 18-39 years, the increasing trend in changes of BMI or waist circumference plateaued after LAN ≥ 20 nW/cm^2^/sr (**Figures 2C, D**).

**Figure 3:**
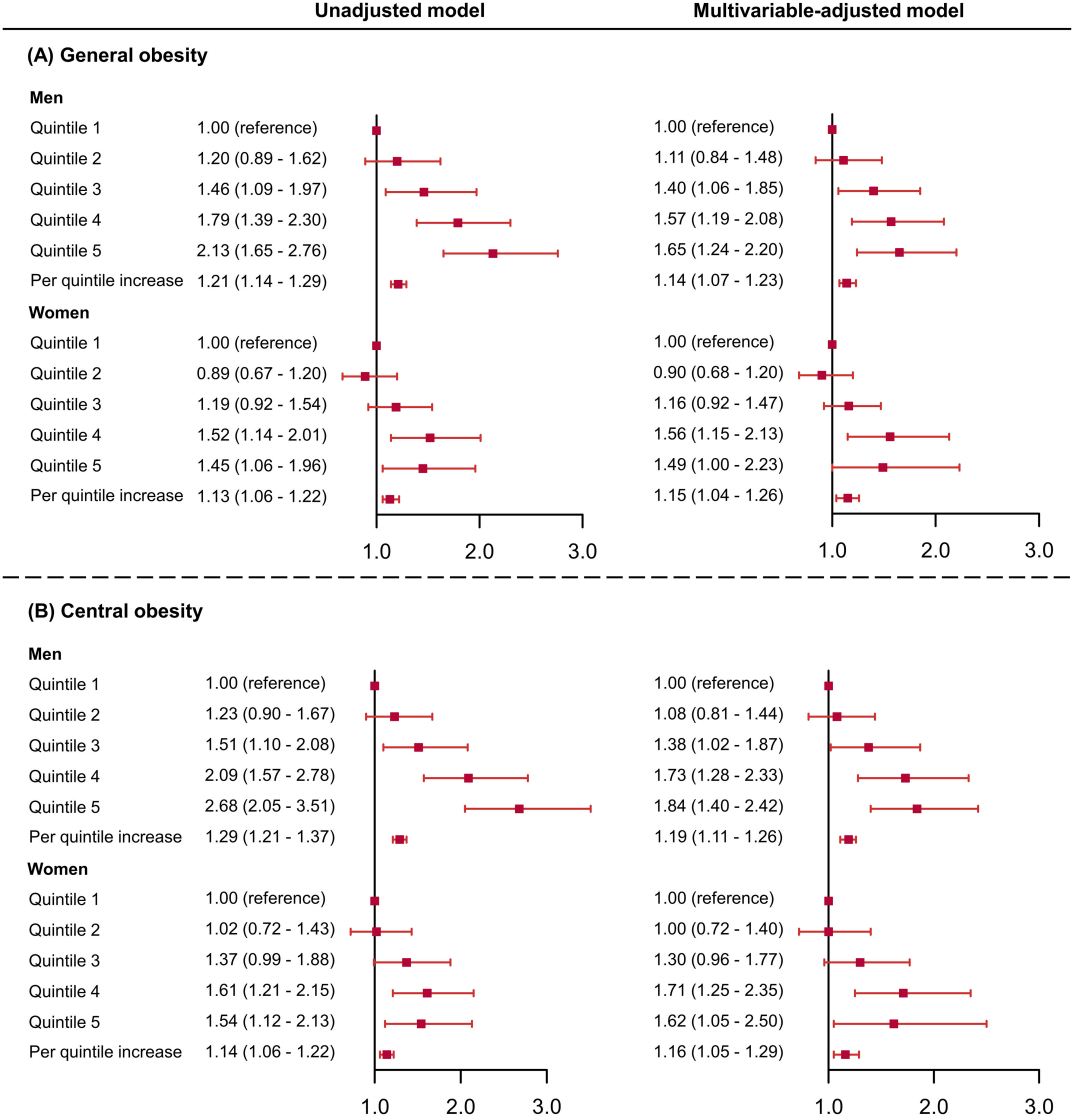
Odds ratios and 95% confidence intervals of the associations between outdoor LAN exposure with general obesity **(A)** and central obesity **(B)** by sex. The model was adjusted for age, education, physical activity, healthy diet score, smoking status, drinking status, urban or rural areas, household income, and HOMA-IR. HOMA-IR, homeostasis model of insulin resistance.

**Figure 4:**
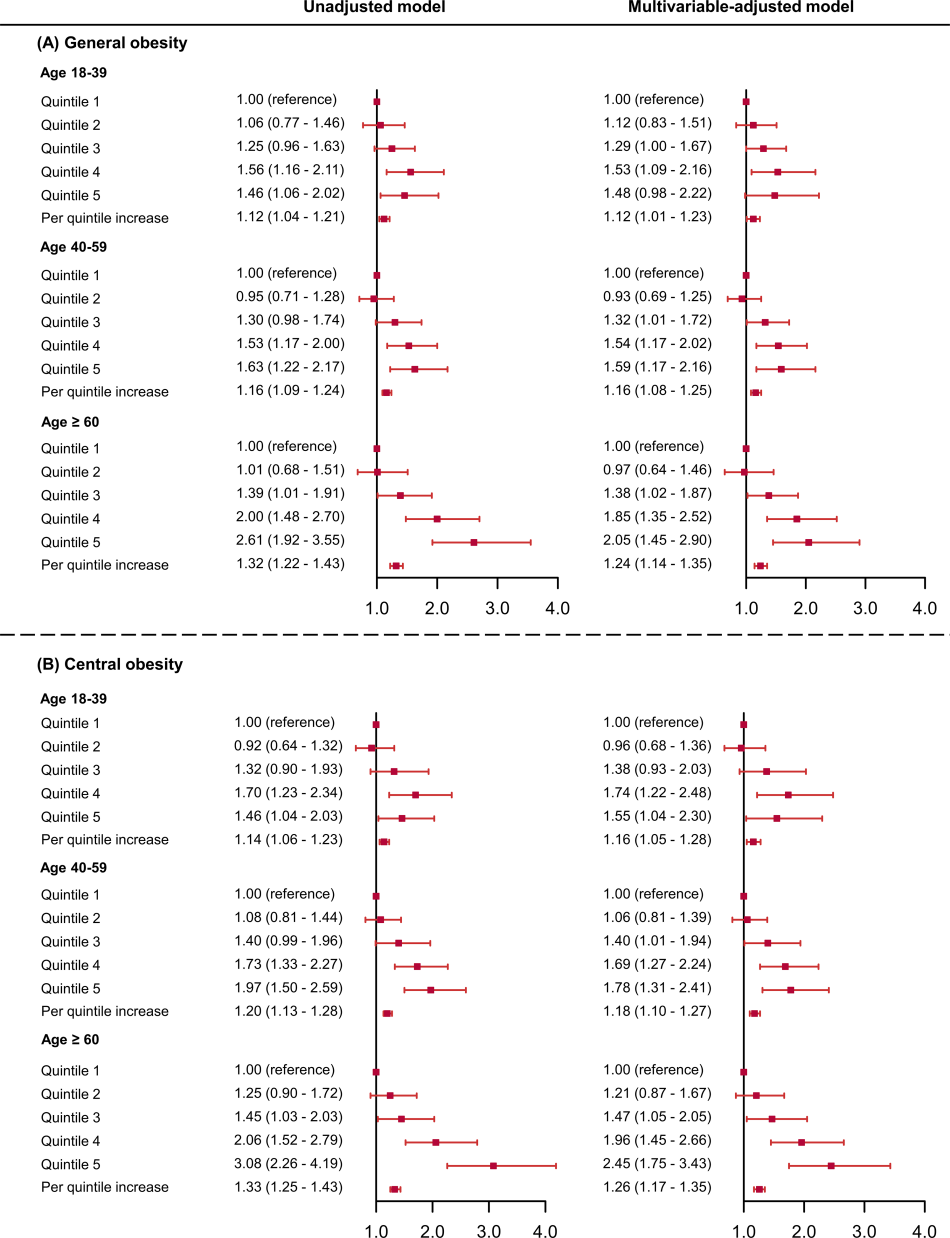
Odds ratios and 95% confidence intervals of the associations between outdoor LAN exposure with general obesity **(A)** and central obesity **(B)** by age groups. The model was adjusted for sex, education, physical activity, healthy diet score, smoking status, drinking status, urban or rural areas, household income, and HOMA-IR. HOMA-IR, homeostasis model of insulin resistance .

In men, outdoor LAN exposure levels in quintile 3 to quintile 5 were significantly associated with higher probabilities of general obesity and central obesity compared with the lowest quintile after adjustment (**Figure 3**). The per 1-quintile increase in the outdoor LAN exposure was significantly associated with general obesity (OR = 1.14, 95% CI = 1.07-1.23) and central obesity (OR = 1.19, 95% CI = 1.11-1.26). In women, the increasing trend in obesity prevalence associated with outdoor LAN exposure quintiles was similar, although not as substantial as in men. In age groups, dose-response relationships were observed for outdoor LAN exposure quintiles and prevalent general obesity, with the trend more substantial in higher age groups (**Figure 4A**). The per 1-quintile increase in the outdoor LAN exposure was significantly associated with general obesity in participants aged 18-39 years (OR = 1.12, 95% CI = 1.01-1.23), 40-59 years (OR = 1.16, 95% CI = 1.08-1.25), and ≥ 60 years (OR = 1.24, 95% CI = 1.14-1.35). Similar findings were observed for central obesity as general obesity in age groups (**Figure 4B**). Using alternative measures of central obesity such as waist-to-height ratio (WHtR) (30) revealed similar findings (**Figures S2, 3**).

## 4 Discussion

Using data from a nationally representative survey of 98,658 Chinese adults from 162 study sites covering 31 provinces across mainland China, we demonstrated that the chronic outdoor LAN exposure exerted significant sex- and age-specific and positive associations on the prevalence of general obesity and central obesity after multivariable adjustment including socioeconomic status. The per 1-quintile increase in the levels of outdoor LAN exposure was associated with a monotonically 10%-30% higher probability of prevalent obesity in each sex- and age- category. Our findings provide epidemiological evidence supporting the adverse effects of LAN on obesity.

Previous population-based studies reported a positive association between LAN and obesity. In a study conducted among Japanese elderly individuals (mean age, 72.8 years), exposure to indoor LAN ≥ 3 lx in an uncontrolled home setting while sleeping was associated with higher ORs for obesity compared with a dim exposure (average < 3 lx) (31). Another epidemiological study investigated over 100,000 women aged 16 years or older in the United Kingdom and used self-reported questionnaires to assess the indoor LAN exposure. They found that BMI, waist to hip ratio, and waist to height ratio increased along with the elevated lightness of LAN in the sleeping room (32). Another study of 43,722 women aged 35 to 74 years in the US also illustrated that exposure to LAN while sleeping might be a risk factor for obesity (33). Studies that used satellite images to assess the level of outdoor LAN exposure had obtained similar findings (24, 34, 35) and one study investigating the association between LAN and obesity using country-level data reported that outdoor LAN contributed to the explanation of 70% of overweight and obesity prevalence (35).

However, the level of outdoor LAN differs nation by nation. While > 99% of the US and European populations live under light-polluted skies (36), China, a developing country with significant differences in economic development at different geographical regions and between rural and urban areas, is exposed to a wide range of outdoor LAN. In addition, few studies have investigated the sex and age differences in the associations of outdoor LAN with obesity. Findings from the current study revealed a monotonically increasing trend in the associations between chronic outdoor LAN exposure and obesity-related parameters including BMI and waist circumference in both sexes. Men seemed to be more susceptible to obesity in response to increased outdoor LAN exposure levels and the sex difference in sleep patterns, behaviors, et al. may play a role. Studies have shown that the visual system in men was more sensitive to light, leading to more significant changes in sleep patterns and that a higher level of LAN exposure was associated with a more substantial possibility of shorter sleep duration in men than women, which conferred a significantly higher risk of future obesity (37–40). Recent experimental studies have shown that circadian misalignment, the potential pathological mechanism of LAN exposure, could lead to higher cravings for energy-dense foods but no changes in energy expenditure in men than women in shift workers (41). Concerning age, although significant associations were found between outdoor LAN and obesity prevalence in all age categories, older adults were more prone to the detrimental effects of LAN exposure on obesity. Studies have elucidated that the prevalence of obesity increased with age but declined slightly in later adulthood (12, 42). Melatonin, the potential regulatory hormone between LAN and obesity, decreases with age. It was reported that older people have lower amounts of brown adipose tissue which could result in more fat accumulation. The presence of brown adipose tissue was positively correlated with night length (43) and prolonged LAN exposure may ‘shorten’ the night length thus might suppress the activity of brown adipose tissue, further synergistically resulting in an imbalance of energy homeostasis and increased fat accumulation (44, 45). In addition, previous studies have found a negative association between outdoor temperature and the activity of brown adipose tissue, indicating that the increase in annual outdoor temperature might lead to higher risks of obesity (46, 47). Therefore, outdoor temperature in the studied areas may also play a part in the association between light at night and prevalent obesity, which warrants further investigation. More studies are needed to illustrate the sex- and age- specific differences and the potential mechanisms in the association between chronic outdoor LAN exposure and obesity.

The major strengths of the current study included the wide coverage of geographical areas across mainland China with a wide range of outdoor LAN exposure levels and the extensive study sample for sex- and age- specific evaluations. The study had several limitations. First, satellite-derived LAN exposure is more likely to represent outdoor lighting levels than indoor LAN exposure at an individual level, leading to misclassification bias. Second, DMSP imaging was not able to provide the specific essence of light including spectrum or wavelength, failing to demonstrate the adverse effect of particular light spectrum. A previous study found that shorter wavelengths of light were more effective than longer wavelengths in suppressing nocturnal melatonin (48). Therefore, more concrete information on LAN could, to a certain extent, promote a better understanding of the relationship between LAN exposure and obesity. Third, data on sleep-related parameters, the timing of food intake, melatonin excretion, psychological status, indoor LAN exposure, outdoor temperature, and alternative measures of central adiposity such as waist-hip ratio were not available. Accordingly, further studies are needed to investigate whether or how sleep patterns, the timing of food intake, melatonin excretion, psychological status, indoor LAN exposure, and outdoor temperature might modify the relationship between LAN and obesity. Fourth, outdoor LAN exposure with multiple years of data leading to the outcome should have been used. However, information of participants’ residence 6 months before the survey or earlier was not recorded, thus LAN exposure before 2010 cannot be determined. Fifth, the cross-sectional design of this study cannot infer causality. Although socioeconomic variables such as education, rural or urban residence, and the household income were adjusted in statistical models, residual confounding cannot be avoided. Finally, the study population included only Chinese. Therefore, findings derived from the current study may have limited generalizability to other populations.

## 5 Conclusion

In conclusion, findings from our study support the role of outdoor LAN exposure in obesity in sex- and age-specific population categories, among which men and older people might be more prone to the deleterious effects of LAN exposure. Prospective studies are warranted to confirm the association and intervention studies might be needed to investigate whether reducing light pollution could be an effective public health strategy for obesity prevention.

## Data availability statement

The datasets presented in this article are not readily available because the data are not publicly available due to privacy protection considering the ethics. Requests to access the datasets should be directed to jane.yuxu@gmail.com.

## Ethics statement

The studies involving human participants were reviewed and approved by the Ethical Review Committee of Chinese Center for Disease Control and Prevention and other participating institutes. The patients/participants provided their written informed consent to participate in this study.

## Author contributions

XZ and RZ: Conceptualization; Formal analysis; Methodology; Writing - original draft. ZX, ZZ, ML, TW, MX, JL, SW, and HL: Data curation; Investigation; Writing - review and editing. WW, GN, and YB: Funding acquisition; Investigation; Resources; Supervision; Writing - review and editing. YC and YX: Conceptualization; Data curation; Methodology; Validation; Writing - review and editing. All authors contributed to the article and approved the submitted version.

## References

[1] Cinzano P Falchi F Elvidge CD. The first world atlas of the artificial night sky brightness. Mon Not Roy Astron Soc (2001) 328(3):689–707. doi: 10.1046/j.1365-8711.2001.04882.x

[2] Navara KJ Nelson RJ. The dark side of light at night: Physiological, epidemiological, and ecological consequences. J Pineal Res (2007) 43(3):215–24. doi: 10.1111/j.1600-079X.2007.00473.x 17803517

[3] Cho Y Ryu S-H Lee BR Kim KH Lee E Choi J. Effects of artificial light at night on human health: A literature review of observational and experimental studies applied to exposure assessment. Chronobiol Int (2015) 32(9):1294–310. doi: 10.3109/07420528.2015.1073158 26375320

[4] Falchi F Cinzano P Elvidge CD Keith DM Haim A. Limiting the impact of light pollution on human health, environment and stellar visibility. J Environ Manage (2011) 92(10):2714–22. doi: 10.1016/j.jenvman.2011.06.029 21745709

[5] Lai KY Sarkar C Ni MY Gallacher J Webster C. Exposure to light at night (LAN) and risk of obesity: A systematic review and meta-analysis of observational studies. Environ Res (2020) 187:109637. doi: 10.1016/j.envres.2020.109637 32497902

[6] Tancredi S Urbano T Vinceti M Filippini T. Artificial light at night and risk of mental disorders: A systematic review. Sci Total Environ (2022) 833:155185. doi: 10.1016/j.scitotenv.2022.155185 35417728

[7] Xiao Q Jones RR James P Stolzenberg-Solomon RZ. Light at night and risk of pancreatic cancer in the NIH-AARP diet and health study. Cancer Res (2021) 81(6):1616–22. doi: 10.1158/0008-5472.CAN-20-2256 PMC8693799 33514513

[8] Sun S Cao W Ge Y Ran J Sun F Zeng Q. Outdoor light at night and risk of coronary heart disease among older adults: A prospective cohort study. Eur Heart J (2021) 42(8):822–30. doi: 10.1093/eurheartj/ehaa846 33205210

[9] Touitou Y Reinberg A Touitou D. Association between light at night, melatonin secretion, sleep deprivation, and the internal clock: Health impacts and mechanisms of circadian disruption. Life Sci (2017) 173:94–106. doi: 10.1016/j.lfs.2017.02.008 28214594

[10] Fonken LK Workman JL Walton JC Weil ZM Morris JS Haim A. Light at night increases body mass by shifting the time of food intake. Proc Natl Acad Sci United States America (2010) 107(43):18664–9. doi: 10.1073/pnas.1008734107 PMC2972983 20937863

[11] Coomans CP van den Berg SAA Houben T van Klinken J-B van den Berg R Pronk ACM. Detrimental effects of constant light exposure and high-fat diet on circadian energy metabolism and insulin sensitivity. FASEB J (2013) 27(4):1721–32. doi: 10.1096/fj.12-210898 23303208

[12] Pan X-F Wang L Pan A. Epidemiology and determinants of obesity in China. Lancet Diabetes Endocrinol (2021) 9(6):373–92. doi: 10.1016/S2213-8587(21)00045-0 34022156

[13] Zheng R Li M Xu M Lu J Wang T Dai M. Chinese Adults are more susceptible to effects of overall obesity and fat distribution on cardiometabolic risk factors. J Clin Endocrinol Metab (2021) 106(7):e2775–e88. doi: 10.1210/clinem/dgab049 33570562

[14] Williams R Periasamy M. Genetic and environmental factors contributing to visceral adiposity in Asian populations. Endocrinol Metab (Seoul) (2020) 35(4):681–95. doi: 10.3803/EnM.2020.772 PMC7803598 33397033

[15] Xiao Z Guo B Gong J Tang Y Shang J Cheng Y. Sex- and age-specific percentiles of body composition indices for Chinese adults using dual-energy X-ray absorptiometry. Eur J Nutr (2017) 56(7):2393–406. doi: 10.1007/s00394-016-1279-9 PMC5602044 27473103

[16] Zhai Y Fang HY Yu WT Wang JZ Yu DM Zhao LY. [Epidemiological characteristics of waist circumference and abdominal obesity among Chinese adults in 2010-2012]. Zhonghua Yu Fang Yi Xue Za Zhi (2017) 51(6):506–12. doi: 10.3760/cma.j.issn.0253-9624.2017.06.010 28592094

[17] Sack RL Lewy AJ Erb DL Vollmer WM Singer CM. Human melatonin production decreases with age. J Pineal Res (1986) 3(4):379–88. doi: 10.1111/j.1600-079x.1986.tb00760.x 3783419

[18] Lachman ME. Adult development, psychology of. In: Smelser NJ Baltes PB, editors. International encyclopedia of the social & behavioral sciences. Pergamon: Oxford (2001). p. 135–9.

[19] Yang G Hu J Rao KQ Ma J Rao C Lopez AD. Mortality registration and surveillance in China: History, current situation and challenges. Popul Health Metr (2005) 3(1):3. doi: 10.1186/1478-7954-3-3 15769298 PMC555951

[20] Xu Y Wang L He J Bi Y Li M Wang T. Prevalence and control of diabetes in Chinese adults. JAMA. (2013) 310(9):948–59. doi: 10.1001/jama.2013.168118 24002281

[21] Lu J Wang L Li M Xu Y Jiang Y Wang W. Metabolic syndrome among adults in China: The 2010 China noncommunicable disease surveillance. J Clin Endocrinol Metab (2017) 102(2):507–15. doi: 10.1210/jc.2016-2477 27898293

[22] Bi Y Jiang Y He J Xu Y Wang L Xu M. Status of cardiovascular health in Chinese adults. J Am Coll Cardiol (2015) 65(10):1013–25. doi: 10.1016/j.jacc.2014.12.044 25766949

[23] National Oceanic and Atmospheric Administration (NOAA). Global radiance calibrated nighttime lights. Available at: https://ngdc.noaa.gov/eog/dmsp/download_radcal.html (Accessed 3, 2022).

[24] Zhang D Jones RR Powell-Wiley TM Jia P James P Xiao Q. A large prospective investigation of outdoor light at night and obesity in the NIH-AARP diet and health study. Environ Health (2020) 19(1):74. doi: 10.1186/s12940-020-00628-4 32611430 PMC7329409

[25] Min JY Min KB. Outdoor light at night and the prevalence of depressive symptoms and suicidal behaviors: A cross-sectional study in a nationally representative sample of Korean adults. J Affect Disord (2018) 227:199–205. doi: 10.1016/j.jad.2017.10.039 29100153

[26] Lloyd-Jones DM Hong Y Labarthe D Mozaffarian D Appel LJ Van Horn L. Defining and setting national goals for cardiovascular health promotion and disease reduction: The American heart association's strategic impact goal through 2020 and beyond. Circulation. (2010) 121(4):586–613. doi: 10.1161/CIRCULATIONAHA.109.192703 20089546

[27] Levy JC Matthews DR Hermans MP. Correct homeostasis model assessment (HOMA) evaluation uses the computer program. Diabetes Care (1998) 21(12):2191–2. doi: 10.2337/diacare.21.12.2191 9839117

[28] National Health Commission of the People’s Republic of China. Criteria of weight for adults (WS/T 428–2013). Beijing: Standards Press of China (2013).

[29] Desquilbet L Mariotti F. Dose-response analyses using restricted cubic spline functions in public health research. Stat Med (2010) 29(9):1037–57. doi: 10.1002/sim.3841 20087875

[30] Yoo E-G. Waist-to-height ratio as a screening tool for obesity and cardiometabolic risk. Korean J Pediatr (2016) 59(11):425–31. doi: 10.3345/kjp.2016.59.11.425 PMC5118501 27895689

[31] Obayashi K Saeki K Iwamoto J Okamoto N Tomioka K Nezu S. Exposure to light at night, nocturnal urinary melatonin excretion, and obesity/dyslipidemia in the elderly: A cross-sectional analysis of the HEIJO-KYO study. J Clin Endocrinol Metab (2013) 98(1):337–44. doi: 10.1210/jc.2012-2874 23118419

[32] McFadden E Jones ME Schoemaker MJ Ashworth A Swerdlow AJ. The relationship between obesity and exposure to light at night: cross-sectional analyses of over 100,000 women in the breakthrough generations study. Am J Epidemiol (2014) 180(3):245–50. doi: 10.1093/aje/kwu117 24875371

[33] Park Y-MM White AJ Jackson CL Weinberg CR Sandler DP. Association of exposure to artificial light at night while sleeping with risk of obesity in women. JAMA Intern Med (2019) 179(8):1061–71. doi: 10.1001/jamainternmed.2019.0571 PMC6563591 31180469

[34] Koo YS Song J-Y Joo E-Y Lee H-J Lee E S.-k. L. Outdoor artificial light at night, obesity, and sleep health: Cross-sectional analysis in the KoGES study. Chronobiol Int (2016) 33(3):301–14. doi: 10.3109/07420528.2016.1143480 26950542

[35] Rybnikova NA Haim A Portnov BA. Does artificial light-at-night exposure contribute to the worldwide obesity pandemic? Int J Obes (Lond) (2016) 40(5):815–23. doi: 10.1038/ijo.2015.255 26795746

[36] Falchi F Cinzano P Duriscoe D Kyba CC Elvidge CD Baugh K. The new world atlas of artificial night sky brightness. Sci Adv (2016) 2(6):e1600377. doi: 10.1126/sciadv.1600377 27386582 PMC4928945

[37] Xiao Q Gee G Jones RR Jia P James P Hale L. Cross-sectional association between outdoor artificial light at night and sleep duration in middle-to-older aged adults: The NIH-AARP diet and health study. Environ Res (2020) 180:108823. doi: 10.1016/j.envres.2019.108823 31627155 PMC6996197

[38] Zhou Q Zhang M Hu D. Dose-response association between sleep duration and obesity risk: a systematic review and meta-analysis of prospective cohort studies. Sleep Breath (2019) 23(4):1035–45. doi: 10.1007/s11325-019-01824-4 30941582

[39] Hedera P Wu D Collins S Lewin JS Miller D Lerner AJ. Sex and electroencephalographic synchronization after photic stimulation predict signal changes in the visual cortex on functional MR images. AJNR Am J Neuroradiol (1998) 19(5):853–7. PMC8337582 9613499

[40] Chellappa SL Steiner R Oelhafen P Cajochen C. Sex differences in light sensitivity impact on brightness perception, vigilant attention and sleep in humans. Sci Rep (2017) 7(1):14215. doi: 10.1038/s41598-017-13973-1 29079823 PMC5660221

[41] Qian J Morris CJ Caputo R Wang W Garaulet M Scheer FAJL. Sex differences in the circadian misalignment effects on energy regulation. Proc Natl Acad Sci United States America (2019) 116(47):23806–12. doi: 10.1073/pnas.1914003116 PMC6876189 31685618

[42] Jura M Kozak LP. Obesity and related consequences to ageing. Age (Dordr) (2016) 38(1):23. doi: 10.1007/s11357-016-9884-3 26846415 PMC5005878

[43] Au-Yong ITH Thorn N Ganatra R Perkins AC Symonds ME. Brown adipose tissue and seasonal variation in humans. Diabetes. (2009) 58(11):2583–7. doi: 10.2337/db09-0833 PMC2768171 19696186

[44] Kooijman S van den Berg R Ramkisoensing A Boon MR Kuipers EN Loef M. Prolonged daily light exposure increases body fat mass through attenuation of brown adipose tissue activity. Proc Natl Acad Sci United States America (2015) 112(21):6748–53. doi: 10.1073/pnas.1504239112 PMC4450411 25964318

[45] Yoneshiro T Aita S Matsushita M Okamatsu-Ogura Y Kameya T Kawai Y. Age-related decrease in cold-activated brown adipose tissue and accumulation of body fat in healthy humans. Obes (Silver Spring Md) (2011) 19(9):1755–60. doi: 10.1038/oby.2011.125 21566561

[46] Ouellet V Routhier-Labadie A Bellemare W Lakhal-Chaieb L Turcotte E Carpentier AC. Outdoor temperature, age, sex, body mass index, and diabetic status determine the prevalence, mass, and glucose-uptake activity of 18F-FDG-detected BAT in humans. J Clin Endocrinol Metab (2011) 96(1):192–9. doi: 10.1210/jc.2010-0989 20943785

[47] Zanobetti A O'Neill MS. Longer-term outdoor temperatures and health effects: A review. Curr Epidemiol Rep (2018) 5(2):125–39. doi: 10.1007/s40471-018-0150-3 PMC6219463 30416932

[48] Haim A Zubidat AE. Artificial light at night: Melatonin as a mediator between the environment and epigenome. Philos Trans R Soc Lond B Biol Sci (2015) 370(1667):20140121. doi: 10.1098/rstb.2014.0121 25780234 PMC4375362

