# Supplementary material for: Sex- and age-specific association between outdoor light at night and obesity in Chinese adults: A national cross-sectional study of 98,658 participants from 162 study sites
Source: Front Endocrinol (Lausanne). 2023 Feb 20;14:1119658. doi: 10.3389/fendo.2023.1119658 (PMC9987422; doi:10.3389/fendo.2023.1119658)
Supplement: Supplementary file 1 [file DataSheet_1.pdf]

Table of contents

**Weighting Methods**	Page 2-3
**Figure S1.** The frequency distribution of participants with different outdoor LAN exposure for (A) men, (B) women, (C) participants with age 18-39 years, (D) participants with age 40-59 years, and (E) participants with age ≥ 60 years	Page 4
**Figure S2.** Associations of outdoor LAN exposure with WHtR categorized by sex (A) and age (B)	Page 5
**Figure S3.** Odds ratios and 95% confidence intervals of the associations between outdoor LAN exposure with obesity defined by WHtR categorized by sex (A) and age (B)	Page 6
**Table S1**. The variance inflation factor of the independent variables in multiple logistic regression for general obesity	Page 7
**Table S2**. The variance inflation factor of the independent variables in multiple logistic regression for central obesity defined by waist	Page 8
**Table S3**. The variance inflation factor of the independent variables in the multiple logistic regression for central obesity defined by WHtR	Page 9
**Table S4**. The prevalence of general obesity and central obesity by quintiles of outdoor LAN exposure	Page 10

Weighting methods

The China Noncommunicable Disease Surveillance Study was designed to select a nationally representative sample of the general population, covering major geographic areas of all 31 provinces, autonomous regions, and municipalities in mainland China. The study recruited participants from 162 study sites within the National Disease Surveillance Point System of Chinese Center for Disease Control and Prevention (China CDC). At each site, a complex, multistage, probability sampling design was used to select participants who were representative of civilian, noninstitutionalized Chinese adults. All the participants were weighted in the sequence of sampling weight, non-response weight, and post-stratification weight. The final weight of each participant was the product of the sampling weight, the non-response weight, and the post-stratification weight.

1. Calculation of sampling weight

Let i be an individual sampling participant. According to the survey design, the following equations were used to calculate the sampling weights.

a. Let w_{si1} be the sampling weight for counties in rural areas or districts in urban areas. It is the reciprocal of the sampling probability of county/district based on the stratified simple random sampling:

$$w_{si1} = \frac{\textit{Total number of counties/districts in the stratum where individual i belongs}}{\textit{Number of sampled counties/districts in the stratum where individual i belongs}}$$

b. Let w_{si2} be the sampling weight for townships in rural areas or subdistricts in urban areas. It is the reciprocal of the sampling probability of township/subdistrict based on the probability proportional to size (PPS) sampling:

$$w_{si2} = \frac{\textit{Population size of the study site where individual i belongs } (Popi1)}{4 \times \text{population size } \textit{of the township/subdistrict where i belongs } (Popi2)}$$

c. Let w_{si3} be the sampling weight for administrative villages in rural areas or neighborhood communities in urban areas. It is the reciprocal of the sampling probability of village/community based on the PPS sampling:

$$w_{si3} = \frac{\textit{Population size of the township/subdistrict where individual i belongs } (Popi2)}{3 \times \text{population size } \textit{of the village/community where individual i belongs } (Popi3)}$$

d. Let w_{si4} be the sampling weight for individuals. It is the reciprocal of the sampling probability of an individual:

$$w_{si4} = \frac{\textit{Number of people } \geq 18 \textit{ years in the village/community where individual i belongs } (Popi3)}{\textit{Number of sampled individuals in the village/community where individual i belongs } (Popi4)}$$

Therefore, the sampling weight for each participant (w_{si}) is:

$$w_{si} = w_{si1} \times w_{si2} \times w_{si3} \times w_{si4} \times \textit{percentage of people aged } \geq 18 \textit{ years in China}$$

2. Calculation of non-response weight

If there are participants who have not responded because they refuse or are not available, non-response weight should be assigned to those who have responded at each stratum. Suppose that the non-response was at random, the non-response weight is calculated as:

$$w_{nr} = \frac{\textit{Planned sample size at each stratum}}{\textit{Number of respondents at each stratum}}$$

3. Calculation of post-stratification weight

Post-stratification weight was assigned to adjust the deviation in important variables of the sampled population from the total Chinese population aged ≥18 years.

a. Definition of sample and population

Sample: sampled participants weighted by sampling weight and non-response weight

Population: Chinese population aged ≥18 years based on the 2010 China population census data

b. There were a total of 156 strata based on stratification variables including sex, age groups, urban or rural areas, and geographical regions.

Table. Stratification variables and number of strata

**Stratification variable**	**Number of strata**	**Description**
Sex	2	Men, women
Age groups	13	18-24, 25-29, 30-34, 35-39, 40-44, 45-49, 50-54, 55-59, 60-64, 65-69, 70-74, 75-79, 80+
Urban or rural areas	2	Urban, rural
Geographical regions	3	East, middle, west

c. Calculation of post-stratification weight

$$w_{pk} = \frac{Population\ size\ at\ stratum\ k}{Weighted\ number\ of\ participants\ at\ stratum\ k}$$

Weight in the above calculation is the product of sampling weight and non-response weight.

4. The final weight

The final weight for each participating individual (individual *i* at stratum *k*) is the product of sampling weight, non-response weight, and post-stratification weight.

$$w_{final} = w_{si} \times w_{nr} \times w_{pk}$$

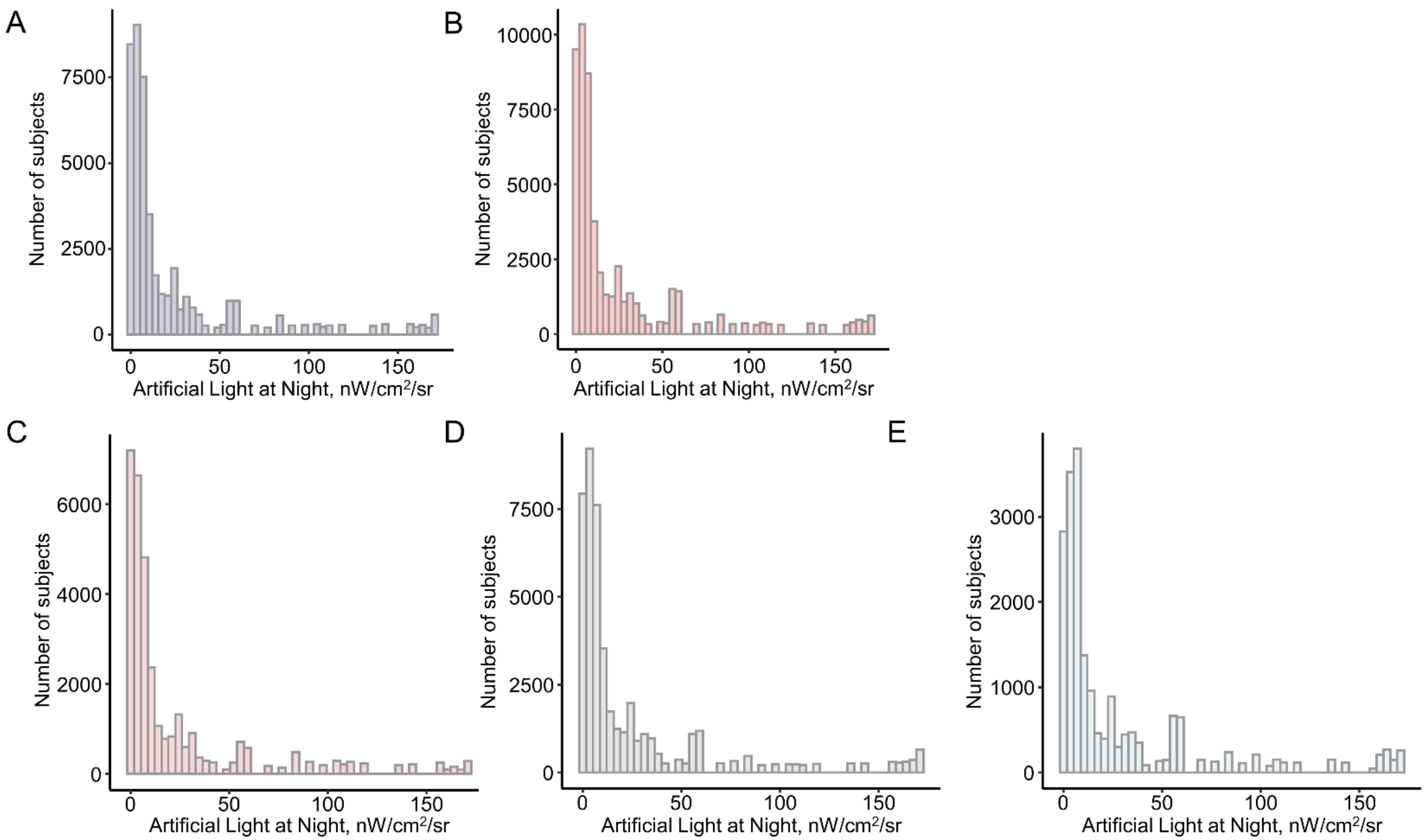

Figure S1. The frequency distribution of participants with different outdoor LAN exposure for (A) men, (B) women, (C) participants with age 18-39 years, (D) participants with age 40-59 years, and (E) participants with age ≥ 60 years.

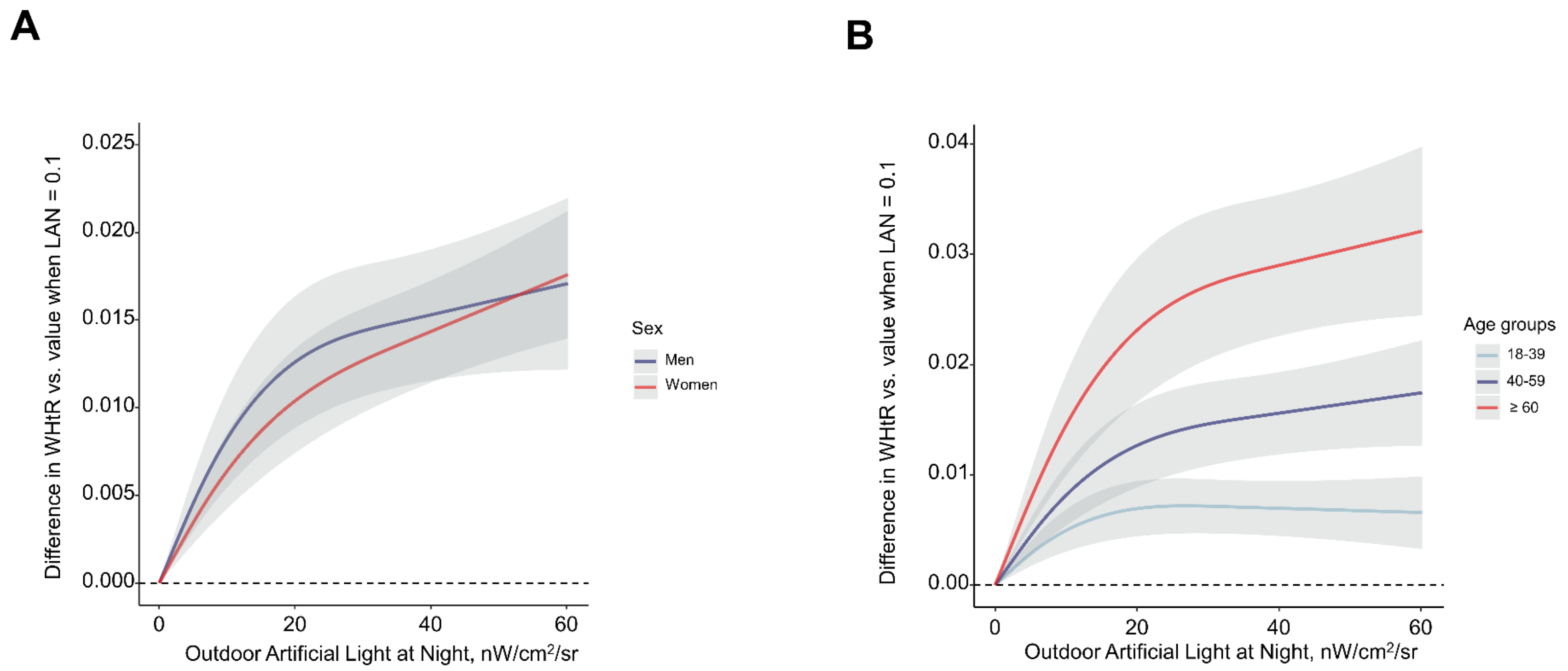

Figure S2. Associations of outdoor LAN exposure with WHtR categorized by sex (A) and age (B).

Light at night was fitted as a smooth term using a restricted cubic spline with 3 knots. The reference outdoor LAN exposure was 0.1 nW/cm^2/sr. Shading areas indicate 95% confidence intervals. Participants within the top right 10% of outdoor LAN exposure were trimmed for the spline model.

The model was adjusted for age (for sex strata), sex (for age strata), education, smoking status, drinking status, physical activity, healthy diet score, urban or rural areas, household income, and HOMA-IR.

BMI, body-mass index; HOMA-IR, homeostasis model of insulin resistance; WHtR, waist-to-height ratio.

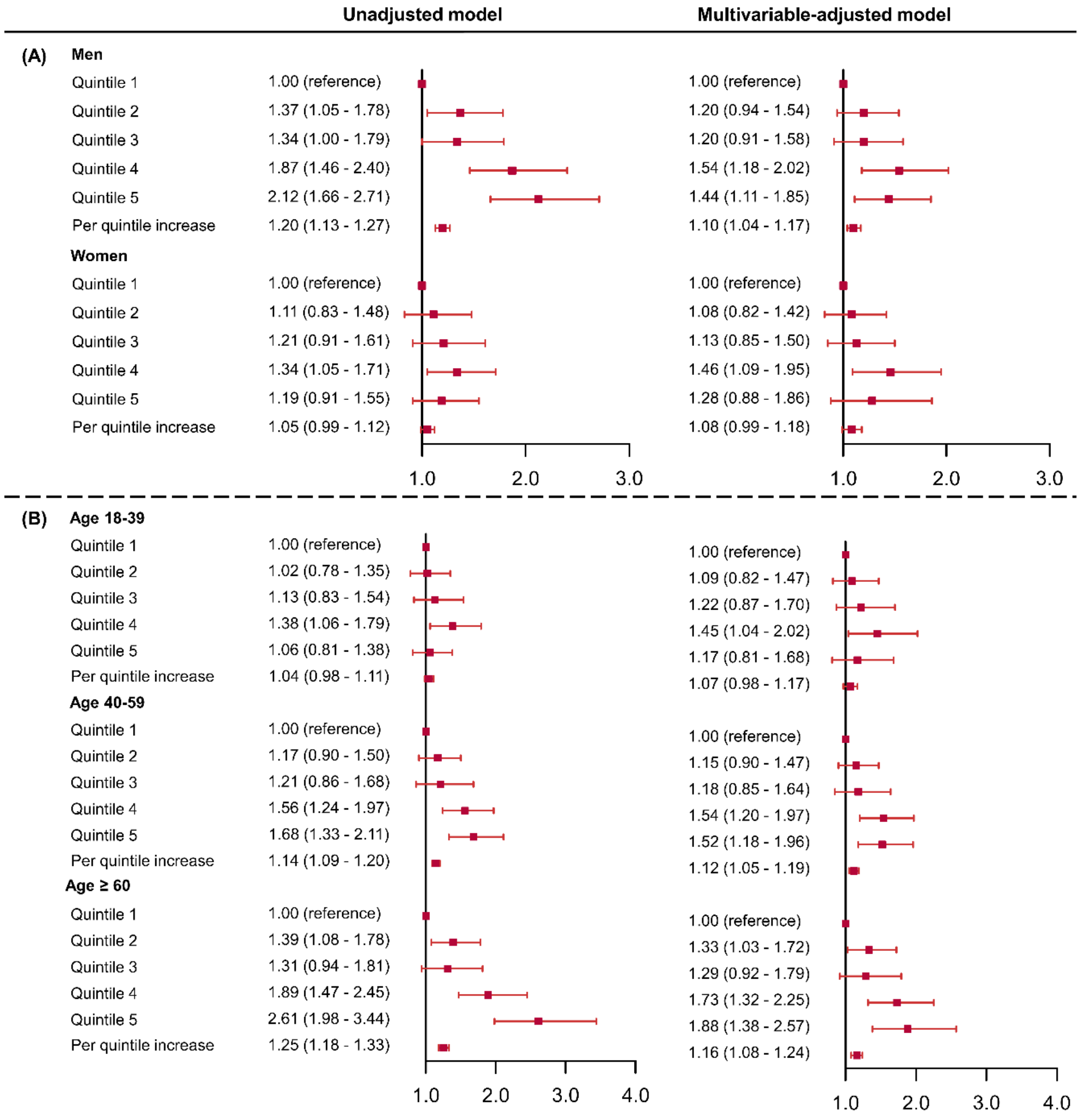

Figure S3. Odds ratios and 95% confidence intervals of the associations between outdoor LAN exposure with obesity defined by WHtR categorized by sex (A) and age (B)†.

The model was adjusted for age (for sex strata), sex (for age strata), education, physical activity, healthy diet score, smoking status, drinking status, urban or rural areas, household income, and HOMA-IR.

† Obesity was defined by waist-to-height ratio ≥ 0.5.

HOMA-IR, homeostasis model of insulin resistance.

Table S1. The variance inflation factor of the independent variables in the multiple logistic regression for general obesity

	Men	Women	Age 18-39 years	Age 40-59 years	Age ≥ 60 years
LAN category	1.790	2.415	2.408	1.625	1.541
Gender	-	-	1.768	1.862	1.722
Age	1.467	1.765	-	-	-
Education	1.697	2.185	1.240	1.912	2.122
Physical activity	1.178	1.270	1.368	1.098	1.082
Smoking	1.388	1.149	1.590	1.623	1.412
Drinking	1.340	1.184	1.825	1.500	1.242
Urban or rural	1.893	2.146	2.289	1.726	1.773
Household income	1.047	1.381	1.328	1.100	1.093
Healthy diet score	1.120	1.474	1.128	1.277	1.495
HOMA-IR	1.100	1.138	1.311	1.250	1.096

The independent variables in models of multiple logistic regression are LAN exposure, age (in gender strata), gender (in age strata), education, smoking status, drinking status, physical activity, healthy diet score, urban or rural areas, household income, and HOMA-IR for each gender or age group.

HOMA-IR, homeostasis model of insulin resistance.

Table S2. The variance inflation factor of the independent variables in the multiple logistic regression for central obesity defined by waist

	Men	Women	Age 18-39 years	Age 40-59 years	Age ≥ 60 years
LAN category	1.512	2.813	2.461	1.888	1.387
Gender	-	-	1.246	1.649	1.385
Age	1.669	1.310	-	-	-
Education	1.578	1.895	1.164	1.306	1.472
Physical activity	1.183	1.409	1.162	1.084	1.125
Smoking	1.350	1.214	1.332	1.494	1.464
Drinking	1.225	1.115	1.599	1.406	1.243
Urban or rural	1.541	2.618	2.231	1.626	1.382
Household income	1.126	1.139	1.172	1.128	1.676
Healthy diet score	1.071	1.231	1.108	1.182	1.145
HOMA-IR	1.486	1.266	1.208	1.371	1.514

The independent variables in models of multiple logistic regression are LAN exposure, age (in gender strata), gender (in age strata), education, smoking status, drinking status, physical activity, healthy diet score, urban or rural areas, household income, and HOMA-IR for each gender or age group.

HOMA-IR, homeostasis model of insulin resistance.

Table S3. The variance inflation factor of the independent variables in the multiple logistic regression for central obesity defined by WHtR†

	Men	Women	Age 18-39 years	Age 40-59 years	Age ≥ 60 years
LAN category	1.708	3.140	3.067	1.658	1.438
Gender	-	-	1.369	1.491	1.543
Age	1.614	1.398	-	-	-
Education	1.746	2.097	1.417	1.570	1.553
Physical activity	1.125	1.207	1.120	1.161	1.272
Smoking	1.271	1.266	1.331	1.251	1.597
Drinking	1.181	1.169	1.344	1.403	1.180
Urban or rural	1.714	2.346	2.501	1.839	1.604
Household income	1.133	1.116	1.095	1.179	1.169
Healthy diet score	1.085	1.305	1.082	1.098	1.398
HOMA-IR	1.859	1.380	1.157	1.635	1.519

The independent variables in models of multiple logistic regression are LAN exposure, age (in gender strata), gender (in age strata), education, smoking status, drinking status, physical activity, healthy diet score, urban or rural areas, household income, and HOMA-IR for each gender or age group.

† Obesity was defined as waist-to-height ratio ≥ 0.5.

HOMA-IR, homeostasis model of insulin resistance.

Table S4. The prevalence of general obesity and central obesity by quintiles of outdoor LAN exposure

	Overall	Men	Women	Age 18-39 years	Age 40-59 years	Age ≥ 60 years
General obesity						
Quintile 1	9.2 (7.6 - 10.8)	8.2 (6.8 - 9.6)	10.2 (8.3 - 12.1)	7.8 (6.4 - 9.3)	11.7 (9.5 - 13.9)	8.2 (6.5 - 9.9)
Quintile 2	9.5 (7.8 - 11.2)	9.7 (7.6 - 11.7)	9.3 (7.5 - 11.0)	8.3 (6.4 - 10.2)	11.2 (9.1 - 13.2)	8.3 (5.8 - 10.8)
Quintile 3	11.7 (9.9 - 13.6)	11.5 (9.2 - 13.9)	12.0 (10.3 - 13.6)	9.6 (8.1 - 11.1)	14.7 (12.2 - 17.2)	11.0 (8.8 - 13.2)
Quintile 4	14.2 (12.1 - 16.3)	13.8 (11.8 - 15.8)	14.7 (12.3 - 17.1)	11.7 (9.4 - 14.0)	16.8 (14.5 - 19.2)	15.1 (12.5 - 17.6)
Quintile 5	15.0 (12.6 - 17.5)	16.0 (13.7 - 18.3)	14.1 (11.4 - 16.9)	11.1 (8.5 - 13.6)	17.7 (14.8 - 20.6)	18.8 (15.6 -22.0)
Central obesity						
Quintile 1	18.0 (14.7 - 21.3)	15.8 (12.8 - 18.9)	20.4 (16.5 - 24.2)	14.3 (11.2 - 17.5)	22.8 (18.9 - 26.6)	21.0 (16.9 - 25.1)
Quintile 2	19.7 (16.5 - 22.8)	18.8 (15.6 - 21.9)	20.6 (16.5 - 24.8)	13.4 (10.5 - 16.2)	24.2 (20.7 - 27.6)	24.9 (21.0 - 28.9)
Quintile 3	24.0 (20.1 - 27.9)	22.2 (18.3 - 26.1)	25.9 (21.7 - 30.1)	18.1 (13.9 - 22.2)	29.1 (23.7 - 34.6)	27.8 (23.2 - 32.5)
Quintile 4	28.7 (25.4 - 31.9)	28.2 (24.6 - 31.7)	29.2 (25.7 - 32.7)	22.1 (18.9 - 25.4)	33.8 (30.2 - 37.4)	35.4 (31.3 - 39.5)
Quintile 5	30.8 (27.1 - 34.6)	33.5 (30.3 - 36.8)	28.3 (23.8 - 32.8)	19.6 (16.3 - 22.9)	36.7 (32.9 - 40.6)	45.0 (40.4 - 49.6)

General obesity was defined as BMI ≥ 28 kg/m^2. Central obesity was defined as waist circumference ≥ 90 cm in men and waist circumference ≥ 85 in women.
The cutoffs of outdoor light at night (LAN) in quintile 1 to quintile 5 are 0 ~ 2.7, 2.8 ~ 5.3, 5.4 ~ 8.7, 8.8 ~ 31.9, 32.0 ~170.8 $nW/cm^2/sr$, respectively.
Data are shown as percentages with 95% confidence intervals.
